# Hepcidin induces HIV-1 transcription inhibited by ferroportin

**DOI:** 10.1186/1742-4690-7-104

**Published:** 2010-12-02

**Authors:** Min Xu, Fatah Kashanchi, Altreisha Foster, Jamie Rotimi, Willie Turner, Victor R Gordeuk, Sergei Nekhai

**Affiliations:** 1 Center for Sickle Cell Disease, Department of Medicine, Howard University, Washington DC 20060, USA; 2 Department of Microbiology, Howard University, Washington DC 20060, USA; 3 National Center for Biodefense and Infectious Diseases, George Mason University, 10900 University Blvd, Manassas, VA 20110, USA

## Abstract

**Background:**

Physiological regulation of cellular iron involves iron export by the membrane protein, ferroportin, the expression of which is induced by iron and negatively modulated by hepcidin. We previously showed that iron chelation is associated with decreased HIV-1 transcription. We hypothesized that increased iron export by ferroportin might be associated with decreased HIV-1 transcription, and degradation of ferroportin by hepcidin might in turn induce HIV-1 transcription and replication. Here, we analyzed the effect of ferroportin and hepcidin on HIV-1 transcription.

**Results:**

Expression of ferroportin was associated with reduced HIV-1 transcription in 293T cells and addition of hepcidin to ferroportin-expressing cells counteracted this effect. Furthermore, exposure of promonocytic THP-1 cells to hepcidin was associated with decreased ferroportin expression, increased intracellular iron and induction of reporter luciferase gene expression. Finally, exposure of human primary macrophages and CD4^+ ^T cells to hepcidin and iron was also associated with induction of viral production.

**Conclusion:**

Our results suggest that the interplay between ferroportin-mediated iron export and hepcidin-mediated degradation of ferroportin might play a role in the regulation of HIV-1 transcription and may be important for understanding of HIV-1 pathogenesis.

## Background

Movement of dietary iron from absorptive enterocytes to portal plasma and of macrophage iron to systemic plasma is mediated by the iron transport protein, ferroportin, and regulated by the hormone, hepcidin, which is synthesized in hepatocytes [1]. Hepcidin binds to ferroportin, and this leads to ferroportin internalization and degradation by lysosomes [1]. Cellular iron is important for HIV-1 transcription, as its removal by iron chelators is associated with inhibition of HIV-1 transcription in cultured cells [2,3].

Several studies suggest that iron stores may influence the course of HIV infection in humans. Increased iron stores correlated with faster HIV-1 progression in HIV-1- positive thalassemia major patients, in HIV-positive patients given oral iron and in HIV-positive subjects with the haptoglobin 2-2 polymorphism [4]. Survival of HIV-positive patients correlated inversely with higher iron stores in bone marrow macrophages [4]. Non-anemic HIV-positive women in Zimbabwe with increased serum ferritin concentration had increased viral load, suggesting that high iron stores may adversely affect HIV infection [5]. Elevated iron predicted higher mortality in Gambian adults infected with HIV-1 [6]. A more recent study showed that both higher and lower iron status correlated with increased mortality in Gambian adults [7]. Different SLC1 (NRAMP1) polymorphisms were also shown to be protective or associated with greater mortality [7].

Experiments by other investigators indicated that, in cultured CEM T cells, excess of iron was associated with increased HIV-1 viral replication, whereas iron chelation with desferrioxamine (DFO) correlated with lower viral replication [8]. Also, the iron chelators, deferoxamine and deferiprone inhibited HIV-1 replication in human primary peripheral blood lymphocytes and macrophages, although the inhibition was attributed to decreased cellular proliferation [9]. Recently, the topical fungicide, ciclopirox, and the iron chelator, deferiprone, were shown to inhibit HIV-1 gene expression at the level of transcription initiation [10]. Both drugs interfered with the hydroxylation step in the hypusine modification of eIF5A [10]. In our own recent studies, the iron chelators, 311 and ICL670, inhibited HIV-1 transcription by inhibiting the cellular activity of cell cycle kinase 2 (CDK2) and by inhibiting phosphorylation of HIV-1 transcriptional activator protein Tat by CDK2 [2]; we previously showed CDK2 to be important for HIV-1 transcription [11]. Our most recent study showed that BpT-based iron chelators, Bp4eT and Bp4aT, prevented association of CDK9 with cyclin T1 and inhibited the activity of the CDK9/cyclin T1 complex [3].

Thus, the studies of others and our own investigation suggest that a decrease in cellular iron might have a negative effect on host HIV-1 gene expression and be protective against HIV-1. In this paper we investigate the effect of the iron exporter, ferroportin, and the ferroportin negative regulator, hepcidin, on HIV-1 transcription and replication in cultured and primary cells. We expressed ferroportin in 293T cells that have undetectable levels of ferroportin and analyzed the effect of ferroportin expression on HIV-1 transcription in the absence and the presence of hepcidin. We proceeded to investigate the effect of ferroportin on HIV-1 in cultured T-cells and monocytes and also in human primary monocytes and CD4+ T cells. Cultured and primary human cells provide a biologically relevant system for the analysis of the effect of ferroportin expression on HIV-1 transcription. Our findings suggest that the interplay between ferroportin expression and its degradation by hepcidin may play a regulatory role in HIV-1 transcription.

## Results

### Expression of ferroportin inhibits HIV-1 transcription

**Figure 1:**
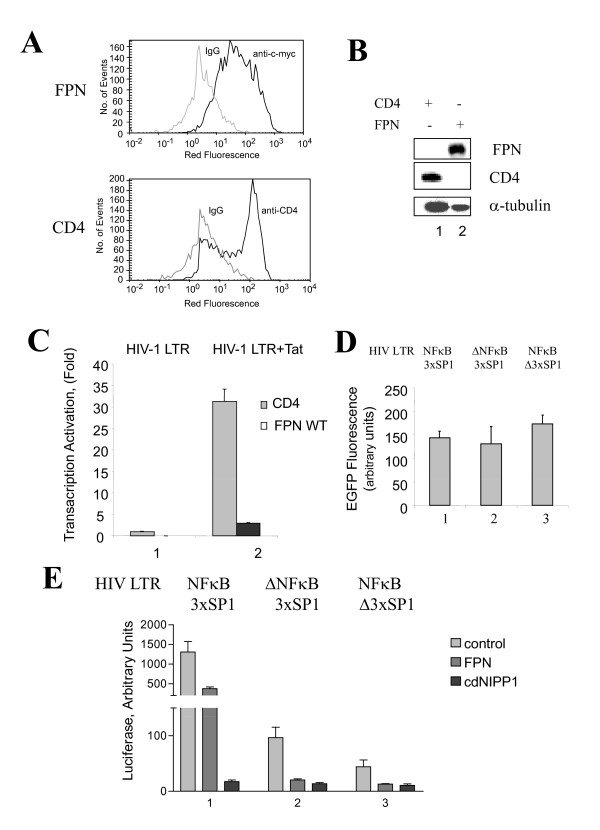
**Expression of ferroportin inhibits HIV-1 transcription**. A and B, Expression of ferroportin in 293T cells. 293T cells were transfected with vectors expressing wild type ferroportin or CD4. At 24 hours posttransfection, the cells were stained with APC-linked antibodies against c-myc or CD4 and analyzed by FACS (A) or the cells were lysed and expression of ferroportin and CD4 was verified by SDS-PAGE and immunoblotting (B). In panel A, solid line - the cells stained with antibodies, shadow line - cells stained with non-specific IgG linked to APC. C, Inhibition of Tat-induced transcription. 293T cells were transfected with vectors expressing CD4 or wild type ferroportin. At 24 hours posttransfection, the cells were re-transfected with HIV-1 LTR*-LacZ *(lane 1) or and HIV-1 LTR*-LacZ *and HIV-1 Tat expression vectors (lane 2) combined with EGFP expression vector. After 24 hours of culturing, the cells were lyzed and β-galactosidase activity was determined using ONPG-based assay. D, Efficiency of transfection verified by co-expression of EGFP. 293T cells were transiently transfected with HIV-1 LTR-Luciferase reporters in combination with CMV-EGFP. The cells were cultured for 24 hrs posttransfection, then lysed and EGFP fluorescence was measured on Luminescence spectrometer. The results are averages of 4 independent transfections. Lane 1, WT HIV-1 LTR (-105 to +77). Lane 2, HIV-1 LTR (-81 to+77) with NF-kB deleted sites. Lane 3, HIV-1 LTR (-105 to +77) with Sp1 inactivated sites. E, Inhibition of basal HIV-1 transcription. 293T cells were transiently transfected with indicated HIV-1 LTR-Luciferase reporters in combination with control CMV-EGFP, ferroportin-EGFP or cdNIPP1-EGFP expression vectors. Lane 1, WT HIV-1 LTR (-105 to +77). Lane 2, HIV-1 LTR (-81 to+77) with NF-κB deleted sites. Lane 3, HIV-1 LTR (-105 to +77) with Sp1 inactivated sites. The cells were cultured for 24 hrs posttransfection, then lysed and the lysates were used to measure GFP fluorescence and luciferase activity.

We expressed ferroportin in 293T cells that express very low levels of endogenous ferroportin [12]. We followed the example of Drakesmith and colleagues [12] who expressed CD8 as a control membrane protein that does not transport iron, except we chose CD4, which participates in HIV-1 viral entry, but has no documented role in HIV-1 transcription. Expression of ferroportin and CD4 was verified by immunofluorescence with anti c-myc (ferroportin) and anti-CD4 antibodies using FACS (Figure 1A) and Western blotting (Figure 1B). To analyze the effect of ferroportin on HIV-1 transcription, the cells were co-transfected with HIV-1 LTR-*LacZ *and CMV-GFP reporters and Tat expression vector. Relative to control cells that expressed CD4, Tat-induced HIV-1 transcription was inhibited in cells that expressed ferroportin (Figure 1C). These results suggest that HIV-1 transcription is negatively affected by the expression of ferroportin.

### Ferroportin expression is associated with inhibition of basal HIV-1 transcription

The HIV-1 promoter contains several binding sites for host transcription factors, including three Sp1 and two NF-κB binding sites [13]. In the absence of Tat, HIV-1 basal transcription is largely regulated by the Sp1 transcription factor [14,15]. Efficiency of transfection was verified by co-expression of EGFP (Figure 1D). Basal, non-Tat-induced activity of the WT HIV-1 LTR promoter was inhibited in 293T cells that expressed ferroportin (Figure 1E, panel 1). As a positive control, we used the PP1 inhibitor, cdNIPP1 (Figure 1E, panel 1), which we previously showed to be a potent inhibitor of Tat-induced and basal HIV-1 transcription [16]. To determine whether the expression of ferroportin has an effect on Sp1-driven or NF-κB-driven HIV-1 transcription, we analyzed the activity of HIV-1 promoters with inactivation of Sp1 sites or deletion of NF-κB sites [17]. Expression of ferroportin was associated with inhibition of the activity of HIV-1 LTR in both settings (Figure 1E, panels 2 and 3). These results indicate that ferroportin expression inhibits basal HIV-1 transcription driven either by Sp1 or NF-kB.

### Hepcidin mediates degradation of ferroportin and is associated with restoration of HIV-1 transcription

**Figure 2:**
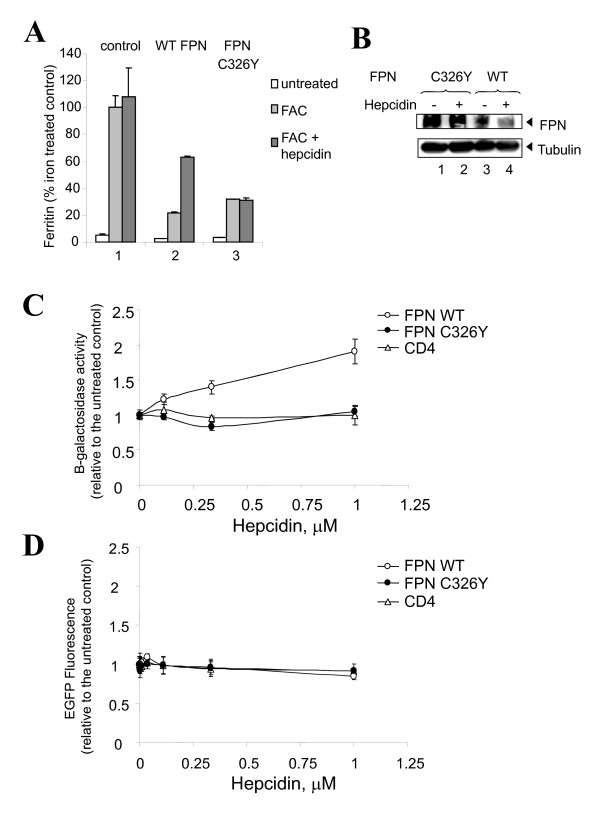
**Hepcidin reverses ferroportin-mediated inhibition of HIV-1 transcription**. A, Ferroportin expression reduces ferritin level in 293T cells. 293T cells were transiently transfected with c-Myc-tagged WT ferroportin-expression vector (panel 2), or mutant c-Myc-tagged C326Y ferroportin -expression vector (panel 3). At 24 hrs posttransfection, the cells were incubated with 20 μM Ferric Ammonium Citrate for 16 hours and then additionally treated with 100 μM cycloheximide for 1 h followed by treatment with 0.3 μM hepcidin for 4 hrs. Ferritin concentration was analyzed by ELISA and normalized to total protein concentration that was determined by Bradford assay. B, Hepcidin reduced ferroportin expression. Protein samples prepared as in panel A were analyzed by SDS-PAGE and Western blotting using c-Myc antibody (upper panel), or tubulin antibodies as loading control (lower panel). C, Hepcidin restores HIV-1 transcription inhibited by ferroportin. 293T cells were transfected with vectors expressing CD4, WT ferroportin or mutant ferroportin C326Y. At 24 hrs posttransfection, the cells were re-transfected with HIV-1 LTR-*lacZ *and HIV-1 Tat expression vectors and immediately treated with indicated concentrations of hepcidin for 24 hrs. After the treatment, the cells were lyzed and β-galactosidase activity was determined using ONPG-based assay. Results are presented relatively to the control that was not treated with hepcidin. D, Hepcidin has no effect on transcription from CMV promoter. 293T cells were transfected with CMV-EGFP vector, treated with hepcidin at 24 hrs posttransfection and incubated another 24 hrs. The cells were lysed and EGFP fluorescence was measured on Luminescence spectrometer. Results are presented relatively to the control that was not treated with hepcidin.

**Figure 3:**
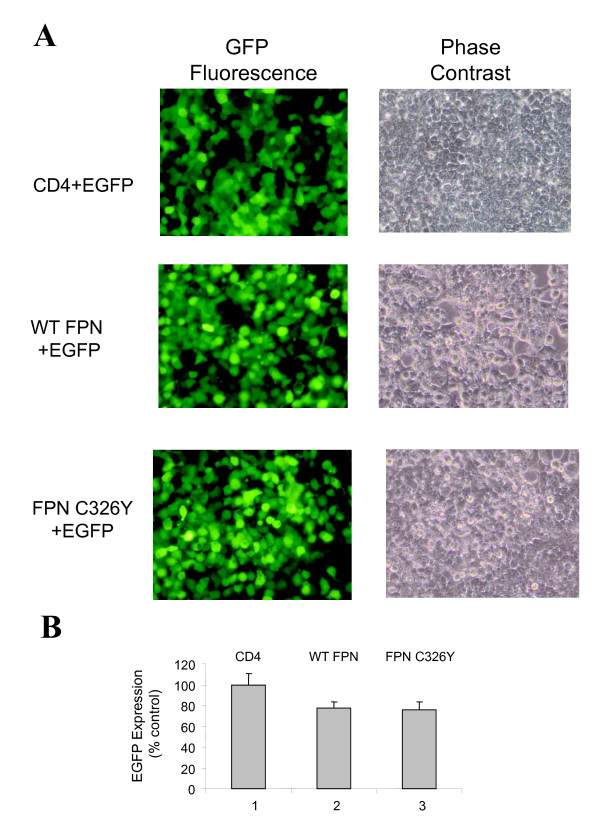
**Ferroportin expression has no effect on transcription from CMV promoter**. 293T cells were transiently transfected with CD4 expression vector, c-Myc-tagged WT ferroportin-expression vector or mutant c-Myc-tagged C326Y ferroportin -expression vector and also co-transfected with CMV-EGFP expression vector. A, photographs of cells on Olympus CKX41 using blue filter for EGFP fluorescence or phase contrast. B, EGFP fluorescence of cell lysates was measured on Luminescence spectrometer.

Treatment of 293T cells with ferric ammonium citrate (FAC) increased cellular ferritin level suggesting an increase in intracellular iron. In keeping with a lack of expression of endogenous ferroportin, exposure to hepcidin did not alter the level of iron achieved with the incubation with FAC (Figure 2A, panel 1). Treatment of 293T cells expressing WT ferroportin or ferroportin C326Y, a mutant that is not sensitive to hepcidin [12], also led to an increase in cellular ferritin. However, in keeping with the iron-exporting function of ferroportin, the magnitude of the increase was less than in the cells not expressing ferroportin (Figure 2A, panels 2 and 3). Treatment of WT ferroportin-expressing cells with hepcidin, followed by treatment with FAC, was associated with a further increase in the level of cellular ferritin (Figure 2A, panel 2). This observation is consistent with the idea that hepcidin reduces ferroportin expression, and is further supported by a control experiment in which ferritin levels were not increased in cells that expressed mutant ferroportin C326Y, which is not sensitive to hepcidin (Figure 2A, panel 3). Analysis of ferroportin expression by immunoblotting showed that hepcidin led to reduced expression of WT, but did not reduce C326Y mutant ferroportin expression (Figure 2B). To analyze whether hepcidin leads to a reversal of the ferroportin-associated inhibition of HIV-1 transcription, 293T cells were transfected with WT ferroportin, mutant ferroportin C326Y or CD4, along with the HIV-1 LTR *LacZ *and Tat -expression vector, and then treated with hepcidin. Addition of hepcidin led to reduced inhibition of HIV-1 transcription by WT ferroportin by a factor of 2, but did not have an effect on HIV-1 transcription in the presence of ferroportin C326Y (Figure 2C). Also, hepcidin had no effect on HIV-1 transcription when CD4 was expressed (Figure 2C). Hepcidin had also no effect on the expression of EGFP (Figure 2D). Expression of CD4, WT ferroportin or ferropotin C326Y did not have an effect on the expression of EGFP from CMV promoter (Figure 3A and 3B). These results suggest that the expression of ferroportin is associated with inhibition of HIV-1 transcription and that the inhibition can be reversed in the presence of hepcidin.

**Figure 4:**
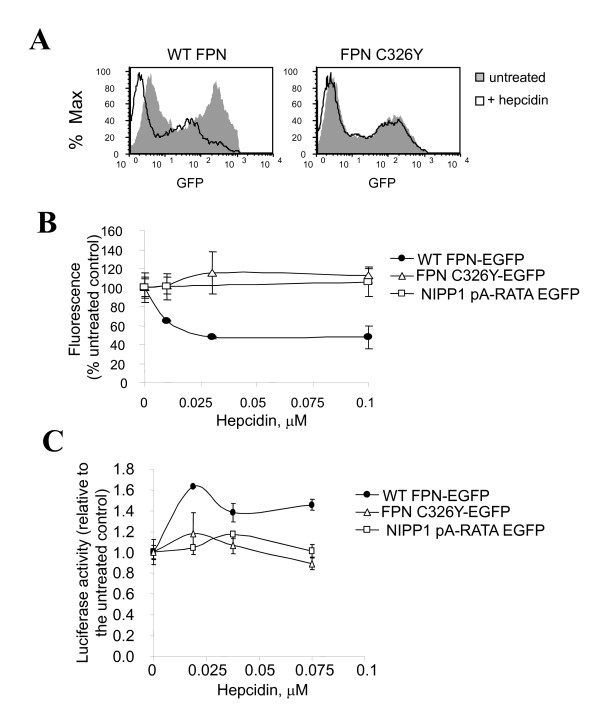
**Hepcidin mediates degradation of ferroportin and partially restores inhibition of HIV-1 transcription**. A and B, Hepcidin mediates degradation of WT but not C326Y ferroportin. 293T cells were transfected with vectors expressing WT ferroportin-EGFP, mutant C326Y ferroportin-EGFP or non-relevant control NIPP1 pA-RATA-EGFP. Transfected cell were treated with FAC for 24 hours and then with 0.5 μM hepcidin (A) or with indicated concentrations of hepcidin (B) for 18 hours. EGFP fluorescence was measured in intact cells using FACS (A) or in cell lysates using Luminescence spectrometer (B). C, Ferroportin reduces inhibition of HIV-1 transcription from HIV-1 proviral DNA by WT ferroportin-EGFP but not mutant C326Y ferroportin-EGFP. 293T cells were transfected with vectors expressing EGFP-fused wild WT ferroportin or mutant ferroportin C326Y and also co-transfected with HIV-1 Luc genomic clone. The cells were treated with FAC for 24 hours and treated with indicated concentrations of hepcidin. After 24 hours of treatment, the cells were lyzed and luciferase activity was determined. EGFP fluorescence was also determined and used to normalize the results.

We further examined the effect of hepcidin on ferroportin using a fusion of ferroportin and green fluorescence protein (EGFP), which allowed us to monitor and quantify the level of ferroportin expression by fluorescence. We subcloned human WT ferropotin and ferroportin C326Y with an EGFP expression vector. Expression of EGFP-fused ferroportin was analysed by FACS which showed that WT ferroportin was sensitive to hepcidin, but ferroportin C326Y was not (Figure 4A). To measure a dose-dependent effect of hepcidin on the expression of ferroportin, 293T cells were transfected with vectors expressing WT ferroportin, mutant ferroportin C326Y, or an inactive mutant of a nuclear inhibitor of protein phosphatase-1 (NIPP1-pA-RATA)-EGFP, as a non-specific control which has no effect on HIV-1 transcription [16]. Addition of hepcidin resulted in a dose-dependent decrease of WT ferroportin expression, but no change in the expression of hepcidin-resistant ferroportin C326Y or NIPP1-pA-RATA EGFP (Figure 4B). Co-transfection of EGFP-fused WT ferroportin or ferroportin C326Y with HIV-1 provirus genomic clone pNL4-3 Luc, which expresses the luciferase reporter cloned in place of Nef [18,19], was associated with an inhibition of luciferase expression (not shown). Treatment of transfected cells with hepcidin reversed the inhibition of HIV-1 Luc in the cells expressing WT ferroportin, but not in cells expressing hepcidin-resistant ferroportin C326Y (Figure 4C). Also, hepcidin had no effect on HIV-1 Luc expression when the cells were co-transfected with NIPP1-pA-RATA EGFP (Figure 4C). These results indicate that ferroportin also inhibited HIV-1 gene expressing from the proviral genomic DNA and that the inhibition was reversed by exposure to hepcidin, which leads to degradation of ferroportin. These observations suggest that the regulation of cellular iron by ferroportin and hepcidin might affect HIV-1 gene expression from an HIV-1 provirus.

### Ferroportin, hepcidin and HIV-1 in cultured monocytes

**Figure 5:**
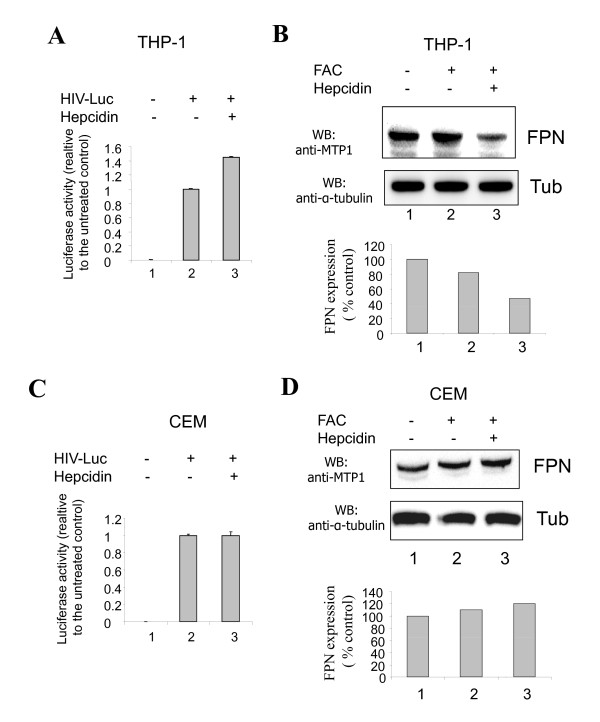
**Expression of ferroportin and effect of hepcidin in cultured THP-1 and CEM cells**. THP-1 cells (A) or CEM T cells (**C**) were treated with FAC for 24 hrs and then treated with 100 μM ascorbic acid for 2 hours. The cells were then infected with VSVG-pdseudotyped HIV-1 Luc virus for 48 hrs (A and C, lanes 2 and 3). Lane 3, the cells were treated with 0.5 μM hepcidin for 6 hours. The cells were lysed, using Luclite buffer system and the Luciferase activity was measured using Labsystems Luminoskan and the data normalization to the protein concentration. THP-1 cells (B) or CEM T-cells (D) were treated with FAC for 24 hrs and then with 100 μM ascorbic acid for 2 hours (lane 2). The cells were then treated with 0.5 μM hepcidin for 6 hours (lane 3). The cell lysates were resolved on 10% SDS-PAGE and immunoblotted with anti-MPT1 antibodies or with anti-tubulin antibodies. Lane 1, untreated control cells.

Next we analyzed the effect of hepcidin on HIV-1 in macrophages, which consume aged red blood cells and play a critical role in recycling or storing the iron derived from the breakdown of hemoglobin. We used cultured promonocytic THP-1 cells as a model system. These cultured cells partially resembled human macrophages due to their expression of CD14 and endogenous ferroportin [20]. As a control, we also used CEM T-cells, a CD4+ human T-lymphoblastoid cell line that is often used for HIV-1 infection and production studies. THP-1 cells and CEM T-cells were pre-treated with ferric ammonium acetate (FAC), followed by ascorbic acid, and then infected with a VSV G-pseudotyped HIV-1-Luc virus that expresses a luciferase reporter (Figure 5). Treatment with hepcidin was associated with increased luciferase activity in THP-1 cells (Figure 5A, panel 3, unpaired t-test P < 0.0001). Analysis of ferroportin expression by immunoblotting in THP-1 cells showed endogenous ferroportin expression, and treatment with hepcidin was associated with decreased expression of ferroportin (Figure 5B). In contrast, hepcidin had no effect on HIV-1 Luc in CEM-T cells (Figure 5C, compare panels 2 and 3, unpaired t-test P = 0.93). Analysis of ferroportin expression in CEM cells showed that endogenous ferroportin was expressed and that its expression was independent of the iron treatment (Figure 5D, lanes 1 and 2); however, treatment with hepcidin did not have an effect on the expression of ferroportin in CEM T-cells (Figure 5D, lane 3). These findings are consistent with the possibility that hepcidin has a moderate inducing effect on HIV-1 transcription in cultured monocytes.

**Figure 6:**
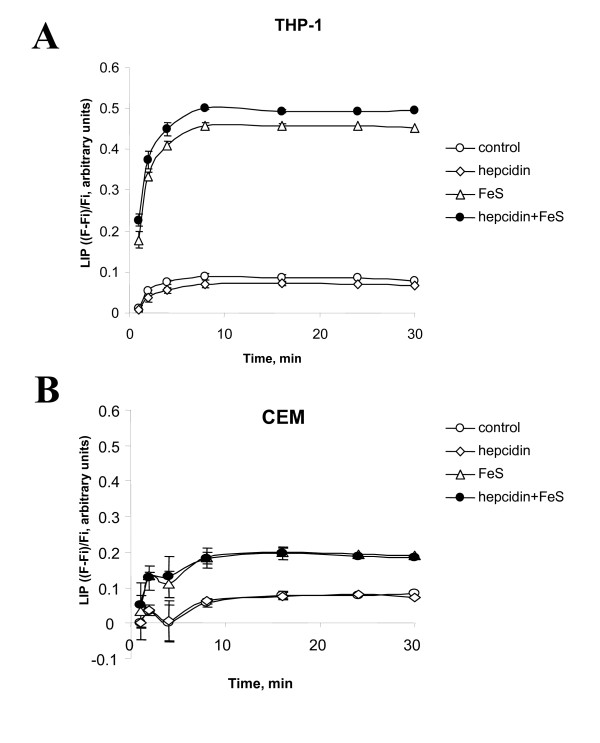
**Hepcidin increases intracellular iron in THP-1 but not in CEM T-cells**. THP-1 cells (A) or CEM T-cells (B) were treated as indicated with 0.5 μM hepcidin for 3 hours and then 25 μM Ferric Sulfate for 1 hr. Then the cells were loaded with 0.1 μM calcein-AM for 10 min at 37°C, and treated with 30 μM SIH. Fluorescence of calcein was measured before and during SIH treatment. Results are presented as fractional fluorescence (***F-Fi)/Fi***, where Fi = fluorescence in the presence of quencher at time 0, and F = fluorescence at given time.

We further analyzed the effect of hepcidin on the levels of labile iron pool (LIP) in THP-1 and CEM cells. We followed the protocol of Cabantchik and colleagues who saturated cells with calcein, a weak iron chelator whose fluorescence is quenched upon binding to iron. Treatment with a strong cell permeable iron chelator, SIH, removes calcein-bound iron, releasing calcein; the increase in fluorescence is measured to determine the amount of chelatable cellular iron [21]. We used the following formula to plot the data: ***F/Fi = 1 + k(Q)*, **where Fi = fluorescence in the presence of quencher at time 0, and F = fluorescence at given time, and Q- concentration of quencher. The (***F-Fi)/Fi ***value is proportional to the concentration of chelatable iron when equilibrium is reached and calcein fluorescence is dequenched. Treatment of THP-1 cells with iron resulted in a significant ~6-fold increase in LIP (Figure 6A). Pre-treatment of THP-1 cells with hepcidin further increased LIP (Figure 6A). The amount of LIP was smaller in CEM T-cells, but the addition of iron resulted also in ~2.5-fold increase in LIP. However, hepcidin had no effect on LIP in these cells (Figure 6B). Taken together, these results suggest that the effect of hepcidin in increasing HIV-1 transcription in THP-1 cells was associated with reduction of ferroportin expression and accumulation of intra-cellular iron.

### Exposure to hepcidin is associated with upregulation of HIV-1 production in primary human mononuclear cells

**Figure 7:**
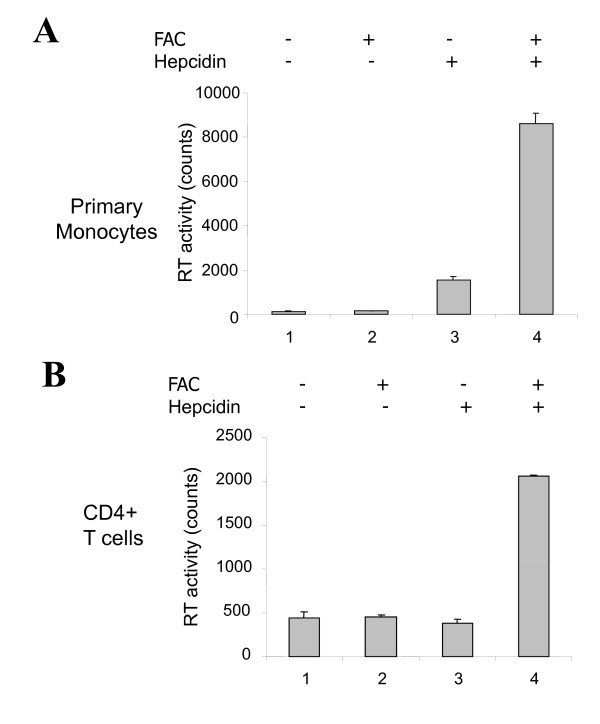
**Hepcidin induces HIV-1 production in primary human monocytes and CD4^+ ^T cells**. Human primary monocytes (A) and human primary CD4+ T cells (B) were infected with dual tropic HIV-1 89.6 strain. At 24 hrs postinfection, the cells were treated with 0.3 μM hepcidin for 3 hrs and then with 100 μM FAC for 24 hrs. At 48 hours postinfection, the medium was collected, and RT was measured.

To investigate the role of hepcidin on HIV-1 in a physiologically relevant cell model, we obtained human primary monocytes and CD4+ T cells from a commercial source. To analyze the effect of hepcidin on HIV-1 replication in these cells, monocytes were differentiated into macrophages, and CD4+ T cells were activated with PHA and IL-2. Then the cells were infected with dual-tropic HIV-1 (89.6), and reverse transcriptase activity (RT) was analysed in media. Treatment with hepcidin was associated with an increase in RT activity in macrophages (Figure 7A, lane 3), and treatment with hepcidin followed by iron significantly induced RT activity (Figure 7A, lane 4). In the infected CD4+ T cells, while treatment with FAC or hepcidin alone did not have an effect on HIV-1 RT activity (Figure 7B, lanes 1-3), treatment of infected cells with hepcidin and FAC together significantly induced HIV-1 RT activity (Figure 7B, lane 4). Thus, hepcidin induced HIV-1 production in primary macrophages and T-cells suggesting that hepcidin expression during HIV-1 infection, along with increased cellular iron, may induce HIV-1 in both T cells and macrophages.

## Discussion

The present study suggests that expression of ferroportin is associated with an inhibitory effect on HIV-1 transcription and that this inhibition can be reversed by hepcidin. Thus the ferroportin/hepcidin interplay may have a role in the regulation of HIV-1 in ferroportin-expressing cells such as monocytes and macrophages as well as, unexpectedly, in T cells.

HIV-1 infection of macrophages has been recognized as an important component of viral pathogenesis [22]. An important function of human macrophages is a recycling of iron to the bone marrow from aged red blood cells, involving iron export by ferroportin [23]. The role of cellular iron in the regulation of HIV-1 replication is not well understood. We recently showed that HIV-1 transcription was inhibited by iron chelators which inhibited cellular activities of the host cell co-activators of HIV-1 transcription, CDK2 and CDK9 [2,3]. The present study shows that increased expression of the iron exporter, ferroportin, is associated with a significant reduction in both basal and Tat-activated HIV transcription. This finding extends our previous observation and suggests that physiological regulators of cellular iron such as ferroportin and hepcidin might be regulators of HIV-1. It is not clear yet if ferroportin or hepcidin has an effect on HIV-1 disease progression. Hepcidin expression is facilitated by IL-6 and other cytokines elevated during inflammation [24], and underlying inflammation is a risk factor for HIV-1 disease progression and pathogenesis [25].

HIV-1 infection of macrophages prolongs their life-time through the induction of PI3K/Akt cell survival pathway [26]. In non-infected macrophages, the PI3K/Akt pathway is negatively regulated PTEN [27]. The PTEN level is lowered by HIV-1 Tat protein in HIV-1 infected macrophages, inducing cell survival [26]. Interestingly, hexamethylene bisacetamide, a potent inducer of cell differentiation and HIV production in chronically infected cells, transiently activates PI3K/Akt pathway; this leads to the phosphorylation of HEXIM1 and the subsequent release of active CDK9/cyclin T1 from its transcriptionally inactive complex with HEXIM1 and 7SK RNA in chronically infected T cell lines and resting CD4+ T-cells [28]. Alkylphospholipds specifically inhibit the activation of Akt kinase activity in HIV-1 expressing macrophages and induce the death of HIV-1 infected macrophages [29]. Because iron deficiency downregulates Akt pathway [30], it is possible that the increase of iron in the hepcidin-treated cells activates Akt pathway and induces HIV-1 transcription.

Iron might be important for different proliferative steps in the life cycle of HIV-1 including reverse-transcription, activation of NF-κB, regulation of HIV-1 transcription, translation of viral mRNA and viral assembly [31]. Previously, Nef, an HIV-1 accessory protein, was shown to down regulate the expression of HFE protein, a modulator of iron homeostasis that is mutated in the iron-overloading disorder hereditary hemochromatosis [32]. Deregulation of HFE by Nef increases iron levels, which coincides with increased HIV-1 gag expression, suggesting a beneficial effect of increased iron on the production of HIV-1 virions and HIV-1 replication [32]. The analysis of HFE deregulation by Nef was carried out in THP-1 cells and also in ex-*vivo *macrophages expressing WT or C282Y HFE [32]. In this study, we used VSV G-pseudotyped HIV-1, which expresses luciferase in place of Nef and therefore lacks the effect of Nef on HFE. It would be of interest to analyze replication of HIV-1 with and without Nef in primary macrophages, with WT and C326Y ferroportin.

Our study showed that HIV-1 transcription is induced in the cells transfected with HIV-1 LTR LacZ expression vector and Tat expression vector. Interestingly, higher concentrations of hepcidin are required for the induction as compared to the induction of transcription from HIV-1 proviral DNA. This may reflect the differences in HIV-1 promoters; HIV-1 genomic DNA contains a full promoter, whereas the HIV-1 LTR LacZ includes only nucleotides -138 to +82 of the HIV-1 genomic DNA. It is possible that induction of HIV-1 transcription by hepcidin involves additional transcriptional factors that act in concert with the HIV-1 Tat protein, such as AP-1 and NFAT [33]. We showed that exposure of differentiated monocytes to hepcidin induced HIV-1 production. Recently, differentiated THP-1 cells were shown to express ferroportin [34,35], and exposure of differentiated THP-1 cells to hepcidin led to a quick degradation of ferroportin [34]. Several ferroportin mutations, including C326Y, that were associated with greater transferrin saturation and more prominent iron deposition in liver *in vivo*, were also resistant to hepcidin. This suggests that these ferroportin mutations continuously export iron, even in the presence of hepcidin [12,36]. Our finding that exposure of the ferroportin C326Y mutant to hepcidin is not associated with enhanced HIV transcription, suggests that other factors that upregulate ferroportin might modulate HIV-1 infection as well. Interestingly, treatment of primary CD4+ T cells with iron and hepcidin augmented the expression of an HIV-1 reporter gene. Thus, our results indicate that the level of ferroportin expression might inversely relate to the level of HIV replication, whereas hepcidin might augment HIV-1 replication. Thus, physiological iron depletion by ferroportin as shown here, iron depletion by iron chelators as we previously showed [2], or reduction of hepcidin expression might restrict HIV-1 replication and be thought of as potential avenues for future HIV-1 therapy. Hepcidin expression, on the other hand, may be associated with augmentation of HIV-1 replication in macrophages and T cells and potential acceleration of HIV-1 progression. Future studies on the effect of hepcidin-resistant mutations in ferroportin and the effect of hepcidin on HIV-1 replication in patients might provide further insights on HIV-1 pathogenesis.

## Conclusion

We are the first to address the effect of ferroportin expression and hepcidin on HIV-1. Collectively, our results indicate that HIV-1 transcription is lower in the cells that express ferroportin and that inhibition of ferroportin expression with hepcidin is associated with augmentation of HIV-1 transcription and viral production.

## Methods

### Materials

Mouse monoclonal anti-ferroportin (MTP-1) antibody was purchased from Alpha Diagnostics. Anti-α-tubulin was purchased from Santa Cruz Biotechnology (Santa Cruz, CA). CEM and 293T cells were purchased from ATCC (Manassas, VA). The Human Promonocytic (THP-1) cells were obtained through the NIH AIDS Research and Reference Reagent Program, Division of AIDS, NIAID, NIH from Drs. Li Wu and Vineet N. KewalRamani [37].

### Cell culture

293T cells were cultured in Dulbecco's Modified Eagles Medium (DMEM) containing 10% fetal bovine serum (FBS) (Life Technologies) and 1% glutamine (Life Technologies) at 37°C in the presence of 5% CO_2_. CEM T cells and human primary monocyte and macrophages (see below) were grown at 37°C in RPMI-1640 medium (Life Technologies) supplemented with 10% fetal bovine serum, 1% glutamine, 1% penicillin and 1% streptomycin. THP-1 cells were grown under the same conditions except that the media was supplemented with 0.05 mM β-mercaptoethanol. The cells were seeded at 0.5 × 10^6 ^cells/ml into 6-well plates.

### Plasmids

Human WT ferroportin and C326Y mutant ferroportin cloned with c-Myc and histidine tags in pcDNA3.1 expression vector was kindly provided Dr. Hal Drakesmith [12]. To generate GFP-tagged human ferroportin, the ferroportin coding sequences from WT ferroportin and ferroportin C326Y mutant were amplified by PCR with primers (forward primer: GC*CTCGAG*ATGACCAGGGCGGGAGATCAC and reverse primer: GC*GGTACC*GTAACAACAGATGTATTTGCTTGATTTTC) that included *XhoI *and *Kpn1 *restriction sites (shown in italic). The PCR products were digested with *XhoI *and *Kpn1 *(BioLabs, Ipswich, MA) and ligated into the pEGFP-N1 vector (Clontech, Mountain View, CA) that was also digested with *XhoI *and *Kpn1 *and ligated. The ligation products were transformed into *E. coli *DH5α cells (Invitrogen, Carlsbad, CA) and kanamycin-resistant colonies were selected. WT ferroportin-EGFP and ferroportin C326Y-GFP-expressing plasmids were purified using Qiagen (Valencia, CA) purification kit and sequenced using Macrogen service (Rockville, MD). The HIV-1 reporter contained HIV-1 LTR (-138 to +82) followed by a nuclear localization signal (NLS) and the LacZ reporter gene (courtesy of Dr. Michael Emerman, Fred Hutchinson Cancer Institute, Seattle, WA) [38]. The pHEF-VSVG expression vector (courtesy of Dr. Lung-Ji Chang) and pNL4-3.Luc.R^-^E^- ^(Courtesy of Dr. Nathaniel Landau) were obtained from the NIH AIDS Research and Reference Reagent Program. The *luciferase *reporters under the control of WT HIV-1 LTR (-105 to +77), HIV-1 LTR (-105 to +77) with Sp1-inactivated sites and HIV-1 LTR (-81 to +77) with NF-κB-deleted sites were courtesy of Dr. Manuel López-Cabrera (Unidad de Biología Molecular, Madrid, Spain) [17]. CD4 expression vector was created by cloning CD4 coding sequence under the control of CMV promoter in the Adeno link vector and was a courtesy of Dr. Marina Jerebtsova (Childrens National Medical Center, Washington DC).

### Transfections

293T cells were seeded in 6 well plates to achieve 50% confluence at the day of transfection. The cells were transfected with indicated plasmids using Lipofectamine and Plus reagents (Life Technologies) following manufacturer's protocol. The efficiency of transfection was verified using a plasmid encoding green fluorescent protein. The cells were cultured for 48 hours post-transfection and then analyzed for HIV-1 transcription or ferroportin expression.

### Western blotting

To measure the expression of c-myc tagged ferroportin expression in 293T cell or endogenous ferroportin in CEM and THP-1 cells, the cells were lyzed in ferritin lysis buffer (50 mM Tris-HCl pH 7.5, 150 mm NaCl, 0.5% NP-40 and 5 mm EDTA). Equal amount of protein (30 μg) was supplemented with SDS-loading buffer (50 mM Tris-HCl pH 6.8, 2% SDS, 10% glycerol, 1% β-mercaptoethanol, 0.1% bromophenol blue), heated at 70°C 2 min and separated on 8% polyacrylamide gel (293T cells) or 8% Tris-Tricine gel (CEM and THP-1 cells). Proteins were transferred to PVDF membranes (Millipore, Allen, TX). Membranes were blocked with 5% milk and incubated overnight at 4°C with anti-c-Myc Tag (Upstate) antibodies, then washed, incubated with anti-rabbit horseradish peroxidase linked F(ab')2 fragment (GE Healthcare UK Limited) and analyzed using Super Signal West Pico Chemiluminescent Substrate Kit (Pierce). For a loading control, we used mouse anti-α-tubulin antibodies (Santa Cruz, CA).

### Measurement of cellular ferritin

The cells were lysed in ferritin lysis buffer (50 mM Tris-HCl pH 7.5, 150 mm NaCl, 0.5% NP-40 and 5 mm EDTA) for 10 min at 4°C. Lysates were spun at 10,000 g for 15 min to precipitate nuclear material and organelles. Protein concentration was measured using Bradford assay (Bio-Rad, Hercules, CA). Ferritin concentration was measured using Spectro Ferritin ELISA kit (Ramco Laboratories,TX). Typically, we used for the ELISA about 50 μg of total protein from 293T cell lysate or lysates of non-iron treated THP-1 or primary monocytes or macrophages. For the iron treated monocytes or macrophages, we used lower amounts of protein (0.5-5 μg).

### Preparation of pseudotyped HIV-1 virus expressing Luciferase (VSV G-HIV-1 Luc)

293T cells were grown on 100 mm plates and transfected using Ca-Phosphate method [16] with VSVG-expressing vector (gpHEF-VSVG) and pNL4-3.Luc.R^-^E^- ^molecular clone that contained two nonsense frame shifts in Env and Vpr genes and Luciferase gene cloned in place of *nef *[18,19]. At 72 h posttransfection, the medium was collected, briefly centrifuged at 1,000 g for 10 min; and then the virus was collected by centrifugation at 4°C for 6 h at 14,000 g. The precipitated virus was resuspended in PBS containing 10% glycerol, aliquoted and stored at -70°C.

### Luciferase Assays, EGFP fluorescence measurements and β-galactosidase assays

293T cells transfected with pNL4-3.Luc.R-E- plasmid or VSVG-HIV-1 Luc infected THP-1 cells or primary monocytes were washed with PBS, resuspended in 100 μl of PBS/well in 96-well plate. Then 100 μl of reconstituted Luclite buffer (Luclite kit, Perkin Elmer) were added to each well, and after 10 min incubation the lysates were transferred into the white plates (Perkin Elmer) and luminescence was measured on Labsystems Luminoscan RT (Perkin Elmer). Where indicated, the fluorescence was measured after the measurement of luminescence at 480 nm excitation and 510 nm emission using a Luminescence Spectrometer LS50B (Perkin-Elmer) equipped with a robotic 96-well scanner. The β-galactosidase assays were performed as we previously described [16].

### Measurement of labile iron

We analyzed labile iron pool (LIP) following the protocol of Cabantchik and colleagues who used calcein-AM followed by iron chelators SIH to detect the amount of chelatable cellular iron [21]. Untreated or iron treated CEM or THP-1 cells were supplemented with 0.2 μM calcein-AM (Molecular Probes, Invitrogen) for 10 min at 37°C. Then the cells were washed with PBS, and fluorescence was measured on Luminescence Spectrometer LS50B (Perkin-Elmer) equipped with a robotic 96-well scanner using 495 nm excitation and 515 nm emission. This fluorescence measurement was designated as time zero. Then 30 μM SIH was added and fluorescence was measured at the indicated time points. We used the following formula to plot the data: ***F/Fi = 1 + k(Q)*, **where Fi = fluorescence in the presence of quencher at time 0, and F = fluorescence at given time, and Q- concentration of quencher. Thus, (***F-Fi)/Fi ***value is proportional to the concentration of chelatable iron when equilibrium is reached, and calcein fluorescence is dequenched.

### Infection of human primary monocytes and CD4+ T cells with dual tropic HIV-1

Human primary monocytes and CD4+ T cells were purchased from Astarte Biologics (Redmond, WA). CD4+ T cells were treated with PHA and IL-2 and half a million cells were infected with dual tropic HIV-1 89.6 (AIDS reagent Catalogue, 50 ng of p24 gag antigen). At 24 hrs postinfection, the cells were treated with 0.3 μM hepcidin for 3 hrs and then with 100 μM FAC for 24 hrs. At 48 hours postinfection, medium was collected, and RT was measured. Monocytes were differentiated into Macrophages via incubation in 10 ng/ml M-CSF for 1 week with medium change every 2 days. At day 4, they were infected with 89.6 virus. For RT assays, viral supernatants (10 μl) were incubated in a 96-well plate with RT reaction mixture containing 1× RT buffer (50 mM Tris-HCl, 1 mM DTT, 5 mM MgCl_2_, 20 mM KCl), 0.1% Triton, poly(A) (10^-2 ^U), poly(dT) (10^-2 ^U) and [^3^H]TTP. The mixture was incubated overnight at 37°C and 5 μl of the reaction mix was spotted on a DEAE Filter mat paper (PerkinElmer, Shelton, CT, USA) washed four times with 5% Na_2_HPO_4 _and three times with water, and then dried completely. RT activity was measured in a Betaplate counter (Wallac, Gaithersburg, MD).

### FACS analysis

C-myc linked ferroportin [12] or CD4 were expressed in 293T cells. The cells were treated with 0.4 μM calcein-AM (Invitrogen) for 1 hr to label the cells for detection in the blue fluorescence channel. The cells were scraped, blocked with 5% goat serum (Sigma) and stained with anti-c-Myc antibodies linked to Allophycocyanin (APC) (Cayman Chemical Company) in 1:100 dilution to detect ferroportin or anti-CD4 antibodies linked to APC (BD Bioscience) in 1:100 dilution to detect CD4 at 4°C for 2 hrs. The cells were precipitated by centrifugation and resuspended in the cell suspension buffer (Agilent) and loaded to the cell checkout chip (Agilent). Fluorescence-Activated Cell Sorting (FACS) was performed on the cell checkout chip in 2100 Bioanalyzer (Agilent). For the analysis of EGFP-linked ferroportin, 293T cells were transfected with GFP-tagged human ferroportin, treated where indicated with 0.1 μM hepcidin for 4 hrs and GFP expression was measured by FACS (Becton-Dickinson) and data were analysed by FlowJo software.

## Competing interests

The authors declare that they have no competing interests.

## Authors' contributions

MX analyzed ferritin expression, cloned human ferroportin into the EGFP-expression vector and measured ferroportin expression. She discussed results and participated in writing of the manuscript. FK conducted experiments with primary monocytes and CD4+ T cells infected with dual-tropic HIV-1 and discussed results. AF measured ferroprotin expression, analyzed the effect of ferroportin on HIV-1 transcription in cultured cells and wrote the manuscript. JR purified plasmids, maintained cell cultures, prepared HIV-1 Luc virus and conducted FACS analysis. WT participated in the design and discussion of the study. VRG participated in the study design, discussion of the results and writing of the manuscript. SN performed overall design, general control and coordination of the study, measured the effect of ferroportin on HIV-1 transcription, conducted studies on labile iron, discussed the results and wrote the manuscript. All authors read and approved the manuscript.
